# Anti-VEGF Agents for Ocular Angiogenesis and Vascular Permeability

**DOI:** 10.1155/2012/852183

**Published:** 2011-11-03

**Authors:** Kenichi Kimoto, Toshiaki Kubota

**Affiliations:** Department of Ophthalmology, Oita University, Faculty of Medicine, Hasama-machi, Yufu-shi, Oita 879-5593, Japan

## Abstract

We review articles describing intravitreal injection of anti-VEGF drug trials, while discussing the mechanisms of the action of anti-VEGF antibodies, and also evaluating their outcomes. Intraocular injections of anti-VEGF drug are considered to be an effective treatment for macular edema after retinal vein occlusion, however, recurrent/persistent edema is common. The recent reports may lead to a shift in treatment paradigm for DME, from laser photocoagulation, to newer approaches using anti-VEGF drugs. There have been several well-publicized prospective, randomized studies that demonstrated the efficacy of intravitreal injection of anti-VEGF drugs for patients with AMD. Adjuvant bevacizumab for neovascular glaucoma may prevent further PAS formation, and it is likely to open up a therapeutic window for a panretinal photocoagulation and trabeculectomy. Intravitreal injection of bevacizumab (IVB) results in a substantial decrease in bleeding from the retinal vessels or new vessels during a standard vitrectomy. IVB has also been reported to be effective for inducing the regression of new vessels in proliferative diabetic retinopathy. The use of bevacizumab in stage 4 or 5 retinopahty of permaturity (ROP) is to reduce the plus sign to help reduce hemorrhage during the subsequent vitrectomy. Some authors reported cases of resolution of stage 4 A ROP after bevacizumab injection.

## 1. Introduction

Recent clinical trials regarding the intravitreal injection of anti-VEGF agents (ranibizumab, bevacizumab, pegaptanib, and aflibercept) have shown excellent results in the treatment of angiogenic pathologies including choroidal neovascularization [1–10], macular edema [11–18], proliferative diabetic retinopathy [19–23], and neovascular glaucoma (NVG) [24–32]. Ranibizumab (Lucentis, Genentech, San Francisco), a fragment of a humanized monoclonal antibody against all VEGF isoforms, is beneficial in the treatment of choroidal neovascularization secondary to age-related macular degeneration [1–7]. Bevacizumab (Avastin, Genentech, San Francisco), a humanized recombinant monoclonal IgG antibody that binds and inhibits all VEGF isoforms, has been approved as an adjuvant agent for the treatment of colorectal carcinoma and has also been increasingly used as an off-label therapy in the field of ophthalmology. Pegaptanib (Macugen, Pfizer, New York), a 28-base ribonucleic acid aptamer, covalently linked to two branched 20-kD polyethylene glycol moieties, was developed to bind and block the activity of extracellular VEGF, specifically the 165 amino acid isoform (VEGF165) [7]. Aflibercept (VEGF Trap-Eye, Regeneron, New York; Bayer, Berlin, Germany) is a 115-kDa recombinant fusion protein consisting of the VEGF-binding domains of human VEGF receptors 1 and 2 fused to the Fc domain of human immunoglobulin-G1 [18]. 

Intravitreal injection of anti-VEGF agents has also been reported to be effective for inducing the regression of new vessels in proliferative diabetic retinopathy (PDR) [19, 20, 33, 34] and neovascular glaucoma (NVG) [24–32, 35] and for improving the vascular permeability in macular edema [11–18]. This injection may provide sufficient time to treat the PDR and NVG patients with retinal photocoagulation. In addition, it may also be used as an adjunctive therapy for mitomycin C (MMC) trabeculectomy to treat NVG [34–38]. Bleeding from the retinal vessels or new vessels during a standard vitrectomy after IVB has been reported to occur significantly less frequently than that observed during a standard vitrectomy without bevacizumab therapy [19, 33, 39–41]. Peters et al. [42] reported on the ultrastructural findings in the primate eye after an IVB. They showed choriocapillaris endothelial fenestrations to dramatically decrease after the injection. In a normal eye, the retinal pigment epithelium (RPE) secretes VEGF at its basal side, which is required for the maintenance of the choriocapillaris [43]. The absence of VEGF may cause a loss of endothelial fenestrations [44]. Moreover, topical, subconjunctival, or stromal injections of bevacizumab against corneal neovascularization were also effective and well tolerated [45–47].

We herein review articles describing intravitreal injection of anti-VEGF drug trials, while discussing the mechanisms of the action of anti-VEGF antibodies,and also evaluating their outcomes. 

## 2. Evaluations of the Outcomes of Anti-VEGF Therapy for Macular Edema following Retinal Vein Occlusion (RVO)

The upregulation of VEGF expression was noted to be elevated in the ocular fluids of central retinal vein occlusion (CRVO) patients [48] and VEGF mRNA expression is also upregulated in the inner nuclear layer in human CRVO pathological specimens [49]. Recent studies have demonstrated that increased production of VEGF occurs early in the disease process and is a major contributor to macular edema following CRVO or branch retinal vein occlusion (BRVO) [11–13]. Thus, there is strong rationale for using VEGF antagonists to treat macular edema following RVO.

There have been two large, well-designed (prospective, randomized, sham injection-controlled, double-masked, multicenter), phase III trials in patients with macular edema following CRVO [14, 15] or BRVO [16, 17].

### 2.1. CRUISE Study [14, 15]

In the Ranibizumab for the Treatment of Macular Edema after Central Retinal Vein OcclUsIon Study: Evaluation of Efficacy and Safety (CRUISE) study, the 392 eligible patients were randomized 1 : 1 : 1 to receive 6 monthly intraocular injections of 0.3 mg or 0.5 mg of ranibizumab or sham injections during the 6-month treatment period. After 6 months, all patients with best-corrected visual acuity (BCVA) ≦20/40 or a central foveal thickness (CFT) ≧250 *μ*m were allowed to receive ranibizumab treatment during the 6-month treatment period. The mean change from baseline BCVA letter score at month 6 was 12.7 and 14.9 in the 0.3 mg and 0.5 mg ranibizumab groups, respectively, and 0.8 in the sham group (*P* < 0.0001 for each ranibizumab group versus sham). The percentage of patients who gained ≧15 letters in BCVA at month 6 was 46.2% (0.3 mg) and 47.7% (0.5 mg) in the ranibizumab groups and 16.9% in the sham group (*P* < 0.0001 for each ranibizumab group versus sham). At month 6, significantly more ranibizumab-treated patients (0.3 mg = 43.9%; 0.5 mg = 46.9%) had a BCVA of ≧20/40 compared with sham patients (20.8%; *P* < 0.0001 for each ranibizumab group versus sham), and the CFT had decreased by a mean of 434 *μ*m (0.3 mg) and 452 *μ*m (0.5 mg) in the ranibizumab groups and 168 *μ*m in the sham group (*P* < 0.0001 for each ranibizumab group versus sham). The median percent reduction in the excess foveal thickness at month 6 was 94.0% and 97.3% in the 0.3 mg and 0.5 mg ranibizumab groups, respectively, and 23.9% in the sham group. 

At 12 months, the mean change from the baseline BCVA letter score was 13.9 and 13.9 in the 0.3 mg and 0.5 mg ranibizumab groups, respectively, and 7.3 in the sham/0.5 mg group (*P* < 0.001 for each ranibizumab group versus sham/0.5 mg). The percentage of patients who gained ≧15 letters from the baseline BCVA at month 12 was 47.0% and 50.8% in the 0.3 mg and 0.5 mg ranibizumab groups, respectively, and 33.1% in the sham/0.5 mg group. On average, there was a marked reduction in the CFT after the first as-needed injection of 0.5 mg ranibizumab in the sham/0.5 mg group to the level of the ranibizumab groups, which was sustained through month 12.

Intraocular injections of 0.3 mg or 0.5 mg of ranibizumab provided rapid improvements in the 6-month visual acuity and CFT following CRVO, and treatments with ranibizumab as needed during months 6 through 11 maintained the visual and anatomic benefits achieved by 6 monthly injections, with low rates of ocular and nonocular safety events. After sham injections for 6 months, ranibizumab injections as needed for 6 months resulted in a rapid reduction in the CFT in the sham group to a level similar to that in the two ranibizumab treatment groups, and an improvement in the BCVA, but not to the same level as that in the two ranibizumab groups. This suggests that there may be a visual penalty incurred by delaying ranibizumab injections in patients with macular edema following CRVO.

Intraocular injections of ranibizumab are considered to be an effective treatment for macular edema after CRVO. However, during the observation period, recurrent/persistent edema or BCVA ≦20/40 was common, necessitating an injection of ranibizumab approximately two-thirds of the time in each group. Additional studies are needed to provide longer follow-up of patients with CRVO treated with ranibizumab to determine whether the dependence on injections is reduced over time and whether strategies such as scatter photocoagulation to areas of retinal nonperfusion provide an added benefit.

### 2.2. BRAVO Study [16, 17]

In the RanibizumaB for the Treatment of Macular Edema after BRAnch Retinal Vein Occlusion: Evaluation of Efficacy and Safety (BRAVO) study, 397 eligible patients were randomized 1 : 1 : 1 to receive 6 monthly intraocular injections of 0.3 mg or 0.5 mg of ranibizumab or sham injections during the 6-month treatment period. After 6 months, all patients with best-corrected visual acuity (BCVA) ≦20/40 or CFT ≧250 *μ*m were allowed to receive ranibizumab during the 6-month observation period. Patients could also undergo rescue laser treatment once during the treatment and observation period. 

The mean change from the baseline BCVA letter score at month 6 was 16.6 and 18.3 in the 0.3 mg and 0.5 mg ranibizumab groups and 7.3 in the sham group (*P* < 0.0001 for each ranibizumab group versus sham). The percentage of patients who gained ≧15 letters in BCVA at month 6 was 55.2% (0.3 mg) and 61.1% (0.5 mg) in the ranibizumab groups and 28.8% in the sham group (*P* < 0.0001 for each ranibizumab group versus sham). At month 6, significantly more ranibizumab-treated patients (0.3 mg, 67.9%; 0.5 mg, 64.9%) had a BCVA of ≧20/40 compared with sham patients (41.7%; *P* < 0.0001 for each ranibizumab group versus sham); and the CFT had decreased by a mean of 337 *μ*m (0.3 mg) and 345 *μ*m (0.5 mg) in the ranibizumab groups and 158 *μ*m in the sham group (*P* < 0.0001 for each ranibizumab group versus sham). The median percent reduction in excess foveal thickness at month 6 was 97.0% and 97.6% in the 0.3 mg and 0.5 mg ranibizumab groups and 27.9% in the sham group. More patients in the sham group (54.5%) received rescue grid laser treatment compared with the 0.3 mg (18.7%) and 0.5 mg (19.8%) ranibizumab groups.

 At 12 months, the mean change from the baseline BCVA letter score was 16.4 and 18.3 in the 0.3 mg and 0.5 mg ranibizumab groups, respectively, and 12.1 in the sham/0.5 mg group (*P* < 0.01, each ranibizumab group versus sham/0.5 mg). The percentage of patients who gained ≧15 letters from the baseline BCVA at month 12 was 56.0% and 60.3% in the 0.3 mg and 0.5 mg groups, respectively, and 43.9% in the sham/0.5 mg group. On average, there was a marked reduction in the CFT after the first as-needed injection of 0.5 mg ranibizumab in the sham/0.5 mg group, which was sustained through month 12. No new ocular or nonocular safety events were identified.

Intraocular injections of 0.3 mg or 0.5 mg of ranibizumab provided a rapid improvement in the 6-month visual acuity and macular edema following BRVO, and treatments with ranibizumab as needed during months 6 through 11 maintained the visual and anatomic benefits achieved by 6 monthly injections, with low rates of ocular and nonocular safety events. In the sham group, treatment with ranibizumab as needed for 6 months resulted in a rapid reduction of the CFT to a similar level as that in the 0.3 mg ranibizumab group and an improvement in the BCVA, but not to the extent of that in the two ranibizumab treatment groups. This suggests that prolonged edema resulting from undertreatment, due to either an as-needed (PRN = pro re nata) dosing schedule or a delay in the initiation of treatment, may result in irreversible retinal damage. Intraocular injections of ranibizumab are considered to be an effective treatment for macular edema after BRVO. However, at 12 months, some patients in each of the groups had a CFT **> **250 *μ*m (16.4% in the 0.3 mg group, 13.7% in the 0.5 mg group, and 21.2% in the sham/0.5 mg group), indicating that the resolution of edema was not universal. Future therapies that address the vein blockage or reduce VEGF production in the affected retina may be necessary to reduce the need for ongoing injection therapy in many patients. Until that time, the mainstay of treatment for macular edema following BRVO is likely to involve frequent intraocular anti-VEGF injections with or without grid laser photocoagulation.

## 3. Evaluations of the Outcomes of Anti-VEGF Therapy for Diabetic Macular Edema

Diabetic macular edema (DME) is the most common cause of moderate vision loss in patients with diabetic retinopathy [50–52]. The pathogenesis of DME is not completely understood, but hyperglycemia- and hypoxia-induced VEGF release are contributing factors [53, 54]. The injection of VEGF into mouse eyes causes the breakdown of the inner blood-retinal barrier [55], and sustained release of VEGF in the eyes of monkeys causes macular edema [56]. The intravitreal VEGF levels have been shown to be elevated in patients with DME [48]. There have been several prospective, randomized studies that have demonstrated the efficacy of intravitreal injection of anti-VEGF drugs (ranibizumab and aflibercept; VEGF Trap-Eye) for patients with DME [18, 57–60].

### 3.1. The RESOLVE Study [57]

This study was a 12-month, multicenter, sham controlled, double-masked study (age > 18 years, type 1 or 2 diabetes, central foveal thickness (CFT) **> **300 *μ*m, and BCVA of 73–39 ETDRS letters) with eyes randomly assigned to intravitreal ranibizumab (0.3 or 0.5 mg; *n* = 51 each) or sham (*n* = 49) treatment. The treatment schedule comprised three monthly injections, after which treatment could be stopped/reinitiated with an opportunity for rescue laser photocoagulation. After month 1, dose doubling was permitted (protocol-defined criteria, the injection volume increased from 0.05 to 0.1 mL and remained at 0.1 mL thereafter). The efficacy (BCVA and CFT) and safety were compared between the pooled ranibizumab and sham arms using the full analysis set (*n* = 151, patients receiving **≧**1 injection). At month 12, the mean ± SD BCVA improved from baseline by 10.3 ± 9.1 letters with ranibizumab and declined by 1.4 ± 14.2 letters with sham treatment (*P* < 0.0001). The mean CFT reduction was 194.2 ± 135.1 *μ*m with ranibizumab and 48.4 ± 153.4 *μ*m with sham treatment (*P* < 0.0001). A gain of **≧**10 letters BCVA from baseline occurred in 60.8% of the ranibizumab and 18.4% of sham eyes (*P* < 0.0001). This phase II randomized multicenter study demonstrated that ranibizumab monotherapy was well tolerated and significantly more effective than sham treatment (with rescue laser) in providing rapid and continuous improvements in the BCVA over 12 months (mean BCVA letter score change from baseline to month 12, +10.3 for ranibizumab versus **−**1.4 for sham; *P* < 0.0001).

### 3.2. The RESTORE Study [58]

The RESTORE study was a 12-month, double-masked, multicenter, laser-controlled, phase III study where 345 eligible patients from 73 centers were randomized 1 : 1 : 1 to 1 of the 3 treatment arms: intravitreal ranibizumab (0.5 mg) injection + sham laser, adjunctive administration of intravitreal ranibizumab (0.5 mg) injection + active laser, or laser treatment + sham injections for 12 months.

Ranibizumab alone and combined with laser treatment was superior to laser monotherapy in improving the mean average change in the BCVA letter score from baseline to months 1 through 12 (+6.1 and +5.9 versus **−**0.8; both *P* < 0.0001). At month 12, a significantly greater proportion of patients had a BCVA letter score of ≧15 and a BCVA letter score level **>**73 (20/40 Snellen equivalent) with ranibizumab (22.6% and 53%, resp.) and ranibizumab **+ **laser treatment (22.9% and 44.9%) versus laser treatment alone (8.2% and 23.6%). The mean CFT was significantly reduced from baseline with ranibizumab (**−**118.7 *μ*m) and ranibizumab **+ **laser (**−**128.3 *μ*m) versus the laser alone (**−**61.3 *μ*m; both *P* < 0.001). Patients received *∼*7 (mean) ranibizumab/sham injections over 12 months.

### 3.3. READ-2 Study [59, 60]

The Ranibizumab for Edema of the MAcula in Diabetes-2 (READ-2) study was designed to compare ranibizumab with focal/grid laser treatment or a combination of both in DME patients. A total of 126 patients with DME were randomized 1 : 1 : 1 to receive 0.5 mg of ranibizumab at baseline and months 1, 3, and 5 (group 1, 42 patients), focal/grid laser photocoagulation at baseline and at month 3 if needed (group 2, 42 patients), or a combination of 0.5 mg of ranibizumab and focal/grid laser treatment at baseline and month 3 (group 3, 42 patients). Month 6 was the primary end point of this study. Starting at month 6, if the retreatment criteria were met, all subjects could be treated with ranibizumab. At month 6, the mean gain in the BCVA was significantly greater in group 1 (+7.24 letters, *P* < 0.01, analysis of variance) compared with group 2 (+0.43 letters), and group 3 (+3.80 letters) was not statistically different from group 1 or 2. For patients with data available at 6 months, improvement of 3 lines or more occurred in 8 of 37 (22%) patients in group 1 compared with 0 of 38 (0%) in group 2 (*P* < 0.002, Fisher exact test) and 3 of 40 (8%) in group 3. The excess foveal thickness was reduced by 50%, 33%, and 45% in groups 1, 2, and 3, respectively. After the primary end point at month 6, most patients in all three groups were treated only with ranibizumab, and the mean number of injections was 5.3, 4.4, and 2.9 during the 18-month follow-up period in groups 1, 2, and 3, respectively. For the 33 patients in group 1, 34 patients in group 2, and 34 patients in group 3 who remained in the study for 24 months, the mean improvement in the BCVA was 7.4, 0.5, and 3.8 letters at the 6-month primary end point, compared with 7.7, 5.1, and 6.8 letters at month 24, and the percentage of patients who gained 3 lines or more of the BCVA was 21%, 0%, and 6% at month 6, compared with 24%, 18%, and 26% at month 24. The percentage of patients with a 20/40 or better Snellen equivalent at month 24 was 45% in group 1, 44% in group 2, and 35% in group 3. The mean CFT, defined as the center subfield thickness, at month 24 was 340 *μ*m, 286 *μ*m, and 258 *μ*m for groups 1, 2, and 3, respectively, and the percentage of patients with center subfield thickness of 250 *μ*m or less was 36%, 47%, and 68%, respectively.

Intraocular injections of ranibizumab provided benefit for patients with DME for at least 2 years, and when combined with focal or grid laser treatments, the amount of residual edema was reduced, as were the frequency of injections needed to control the edema.

### 3.4. The DA VINCI Study [18]

The primary purpose of the DME and VEGF Trap-Eye: Investigation of Clinical Impact (DA VINCI) Study (multicenter, randomized, double-masked, phase 2 clinical trial) was to determine whether different doses and dosing regimens of intravitreal VEGF Trap-Eye are superior to standard macular laser treatment over a 24-week study in eyes with DME. 

VEGF Trap-Eye is a 115-kDa recombinant fusion protein consisting of the VEGF-binding domains of human VEGF receptors 1 and 2 fused to the Fc domain of human immunoglobulin-G1. Animal studies have demonstrated that intravitreal VEGF Trap-Eye has theoretic advantages over ranibizumab and bevacizumab, including a longer half-life in the eye and a higher binding affinity to VEGF-A. In addition, the fusion protein binds placental growth factors 1 and 2, which have been shown to contribute to excessive vascular permeability and retinal neovascularization.

A total of 221 patients were assigned to 1 of 5 treatment regimens: 0.5 mg VEGF Trap-Eye every 4 weeks; 2 mg VEGF Trap-Eye every 4 weeks; 2 mg VEGF Trap-Eye for 3 initial monthly doses and then every 8 weeks; 2 mg VEGF Trap-Eye for 3 initial monthly doses and then on a PRN basis; or macular laser photocoagulation. Patients in the 4 VEGF Trap-Eye groups experienced mean visual acuity benefits ranging from **+**8.5 to **+**11.4 ETDRS letters versus only **+**2.5 letters in the laser group (*P* < 0.0085 for each VEGF Trap-Eye group versus laser). Gains from baseline of +0, +10, and +15 letters were seen in up to 93%, 64%, and 34% of VEGF Trap-Eye groups versus up to 68%, 32%, and 21% in the laser group, respectively. The mean reductions in the CFT in the 4 VEGF Trap-Eye groups ranged from **−**127.3 to −194.5 *μ*m compared with only **−**67.9 *μ*m in the laser group (*P* < 0.0066 for each VEGF Trap-Eye group versus laser). VEGF Trap-Eye was generally well tolerated. The ocular adverse events in patients treated with VEGF Trap-Eye were generally consistent with those seen with other intravitreal anti-VEGF agents.

This study demonstrated that the different doses (0.5 or 2 mg) and dosing regimens (given every 4 weeks, every 8 weeks, or on a PRN basis) of VEGF Trap-Eye were all individually superior to laser therapy and resulted in statistically significant increases in visual acuity and reductions in retinal thickness at week 24. The reports mentioned above may lead to a shift in treatment paradigm for DME, from laser photocoagulation, to newer approaches using either ranibizumab or aflibercept.

## 4. Evaluations of the Outcomes of Anti-VEGF Therapy for Age-Related Macular Degeneration

There have been several well-publicized prospective, randomized studies that have demonstrated the efficacy of intravitreal injection of anti-VEGF drugs (ranibizumab, pegaptanib, and aflibercept; VEGF Trap-Eye) for patients with age-related macular degeneration (AMD).

 The MARINA (Minimally classic/occult trial of the Anti-VEGF antibody Ranibizumab In the treatment of Neovascular Age-related Macular Degeneration) study [1] demonstrated the intravitreal administration of ranibizumab for 2 years to prevent vision loss while improving the mean visual acuity with low rates of serious adverse events, in patients with minimally classic or occult choroidal neovascularization secondary to age-related macular degeneration.

The ANCHOR (ANti-VEGF antibody for the treatment of predominantly classic CHORoidal neovascularization in age-related macular degeneration) study [2] demonstrated that ranibizumab provided greater clinical benefit after 2 years than verteporfin PDT in patients with age-related macular degeneration with new-onset, predominantly classic CNV.

The PIER (Phase IIIb, multicenter, randomized, double-masked, sham Injection-controlled study of the Efficacy and safety of Ranibizumab in subjects with subfoveal CNV with or without classic CNV secondary to age-related macular degeneration) study [3, 4] demonstrated that ranibizumab administered monthly for three months and then quarterly provided VA benefits to patients with neovascular AMD and was well tolerated. However, the observations from the MARINA and ANCHOR trials suggest that the PIER regimen of dosing every three months after three monthly doses provides less benefit in terms of VA on average than continued monthly dosing. Monthly dosing may be necessary in some patients to achieve maximal treatment benefit from ranibizumab.

The PrONTO (Prospective Optical coherence tomography imaging of patients with Neovascular age-related macular degeneration Treatment with intraOcular ranibizumab) study [28, 29] used an Optical Coherence Tomography (OCT)-guided variable dosing regimen with intravitreal ranibizumab. During the first year, retreatment with ranibizumab was performed at each monthly visit if any criterion was fulfilled such as an increase in OCT-CFT of at least 100 *μ*m or a loss of 5 letters or more. During the second year, the retreatment criteria were amended to include retreatment if any qualitative increase in the amount of fluid was detected using OCT. This study demonstrated that at month 24, the mean VA improved by 11.1 letters and the CFT decreased by 212 *μ*m. The VA improved by 15 letters or more in 43% of patients. These VA and OCT outcomes were achieved with an average of 9.9 injections over 24 months. As-needed (PRN), OCT-guided variable dosing with intravitreal ranibizumab resulted in VA outcomes comparable to the outcomes from the phase III clinical studies (monthly injection), but fewer intravitreal injections were required.

The V.I.S.I.O.N (VEGF Inhibition Study In Ocular Neovascularization) study [7] demonstrated that in the group given pegaptanib at 0.3 mg, 70% of patients lost fewer than 15 letters of visual acuity, as compared with 55% among the controls (*P* < 0.001).

Pegaptanib appears to be an effective therapy for AMD. However, it does not lead to any improvement in the mean visual acuity.

The LEVEL (EvaLuation of Efficacy and Safety in Maintaining Visual Acuity with SEquential Treatment of NeovascuLar AMD) study [8] assessed the efficacy of pegaptanib as maintenance therapy in AMD patients who experienced a clinical improvement in disease following an induction phase. The induction maintenance using nonselective, followed by selective VEGF inhibitors should be considered for the treatment of AMD. Such an approach has special relevance for patients with cardiovascular comorbidities who require anti-VEGF drugs to manage their AMD.

The Comparison of Age-Related Macular Degeneration Treatments Trials (CATT) research group [9] demonstrated that at 1 year, bevacizumab and ranibizumab had equivalent effects on visual acuity when administered according to the same schedule. Bevacizumab administered monthly was equivalent to ranibizumab administered monthly, with 8.0 and 8.5 letters gained, respectively.

The CLEAR-IT (CLinical Evaluation of Anti-angiogenesis in the Retina Intravitreal Trial) study [10] demonstrated that PRN dosing of VEGF Trap-Eye after 12 weeks of monthly or quarterly fixed dosing maintained clinically and statistically significant improvements in vision and retinal thickness until at least week 52 in patients with neovascular AMD, with a low frequency of reinjection. VEGF Trap-Eye was generally well tolerated, with a safety profile similar to that reported with other intravitreally administered anti-VEGF agents.

## 5. Neovascular Glaucoma

The normal trabecular meshwork consists of trabecular spaces and trabecular cells covering trabecular beams, and it contains no vascular structures. Neovascular glaucoma is a severe consequence of ocular ischemic diseases, such as diabetic retinopathy and central retinal vein occlusion. The elevated intraocular pressure (IOP) is considered to result from an increased permeability of the newly formed vessels [61], angle closure by the peripheral anterior synechia, and intertrabecular neovascular tissue [62, 63]. An ultrastructural study showed that the neovascular tissue in the intertrabecular spaces may be one of the factors responsible for the IOP elevation. The layers of vascular endothelial cells have junctional complexes and fenestrations [63]. The intravitreal injection of bevacizumab was reported to be effective for inducing the regression of new vessels, although its effect seems to be temporary [24–32, 35–38]. In an animal study, bevacizumab penetrated quickly into the iris, anterior chamber angle and ciliary body after IVB. This finding supports the clinically observed rapid effect of bevacizumab in the treatment of iris neovascularization [64]. This injection may provide us with sufficient time to treat these patients with retinal photocoagulation. In addition, it may also be used as an adjunctive therapy for a mitomycin C (MMC) trabeculectomy in order to treat NVG. The use of preoperative IVB may decrease postoperative hyphema, however the beneficial effects of IVB are controversial for surgical outcomes over longer periods [37, 38].

Ishibashi et al. [65] performed anterior segment angiography with both fluorescein (FA) and indocyanine green (IA) using a Heidelberg retina angiograph 2 on the patients with NVG. FA showed intensive leakage of the dye from newly formed vessels, although IA revealed the vessel structures clearly without dye leakage. In both iris and angle angiography, dye leakage with FA was decreased after IVB compared with that before treatment. The vascular structures in the iris and angle observed with IA, however, did not change after IVB, although the newly formed vessels in the tissues seemed to vanish based on slit-lamp examinations.

Kubota et al. [66] showed that CD34 positive vascular endothelial cells formed capillary-like structures in the trabecular meshwork of NVG after an intravitreal injection of bevacizumab. Although the slit lamp and gonioscopic examinations revealed a regression of the newly formed vessels after the intravitreal injection of bevacizumab, they morphologically showed that the vascular endothelial cells were present in the trabecular meshwork. Electron microscopy revealed that the layer of endothelial cells had junctional complexes. However, the fenestrations of the vascular endothelial cells were significantly fewer in the trabecular meshwork of neovascular glaucoma eyes treated with bevacizumab than those without the therapy [66, 67]. The intravitreal injections of bevacizumab resulted in a decrease in VEGF in the aqueous humor [68]. Therefore, it is considered that a decreased VEGF concentration in the aqueous humor causes a reduction of the fenestrations of the vascular endothelial cells in the trabecular tissue of neovascular glaucoma after an intravitreal injection of bevacizumab. These morphological alterations may play an important role in the clinical effects of intravitreal bevacizumab for neovascular glaucoma.

Although the effect of bevacizumab seems temporary, previous clinical reports suggested the potential for intravitreal bevacizumab as a treatment option for neovascular glaucoma [24–32]. Adjuvant bevacizumab for neovascular glaucoma may prevent further PAS formation, and it is likely to open up a therapeutic window for a panretinal photocoagulation and trabeculectomy. Johnson et al. [61] reported that one of the factors affecting the IOP elevation is the concentration of the serum protein in the aqueous humor. It has been suggested that one possible mechanism underlying the IOP elevation is the increased permeability of the newly formed vessels. The fenestrations of the newly formed vessels are thus considered to have a close relationship to such permeability. Bevacizumab may induce a reduction of the capillary structures and fenestrations of the vascular endothelium in the trabecular tissue, and therefore, has an IOP lowering effect and a reduced risk of hemorrhage during trabeculectomy.

It was still unclear from these studies whether bevacizumab temporally induces a reduction of the perfusion of the newly formed vessels, or a disappearance of the newly formed vessels in the trabecular meshwork. Another previous study [67] suggested that the number and perfusion of the newly formed vessels were reduced. However, to fully elucidate how a reduction of the newly formed vessels occurred in the trabecular meshwork of neovascular glaucoma after intravitreal bevacizumab injection, further studies are needed. 

## 6. Proliferative Diabetic Retinopathy

VEGF is a key molecule involved in the development of retinal neovascularization [69–71]. Studies have demonstrated not only a correlation of the VEGF levels with the severity of proliferative diabetic retinopathy (PDR), but also a reduction in the levels after successful laser treatment of PDR [48]. Ischemia in the retina due to microvascular occlusion induces the release of VEGF into the vitreous cavity; highly concentrated VEGF in the ocular fluid leads to the growth of new vessels [48]. VEGF also increases the permeability of capillary vessels and contributes to diabetic macular edema [72, 73]. Retinal fibrovascular membranes, including neovascularization, represent an important risk factor for severe vision loss in patients with diabetic retinopathy. An ultrastructural study of the fibrovascular membranes in a patient with proliferative diabetic retinopathy demonstrated that the newly formed vessels frequently showed endothelial fenestrations. Occasionally, the tight junctions between endothelial cells appeared altered [74]. 

IVB results in a substantial decrease in VEGF in the aqueous humor [68]. Bleeding from the retinal vessels or new vessels during a standard vitrectomy after IVB has been reported to occur significantly less frequently than that observed during a standard vitrectomy without bevacizumab therapy [33, 39–41]. IVB has also been reported to be effective for inducing the regression of new vessels in PDR [19, 20, 33, 34]. An angiographic study demonstrated that the leakage was noted to diminish as early as 24 hours after IVB [19]. IVB also resulted in marked regression of neovascularization and rapid resolution of vitreous hemorrhage [22]. A randomized, placebo-controlled clinical trial revealed the efficacy of IVB in reducing the rate of early postoperative vitreous hemorrhage after vitrectomy in diabetic patients [75]. The balance between connective tissue growth factor and vascular endothelial growth factor may be the strongest predictor of the degree of fibrosis; a shift toward connective tissue growth factor can underlie the aggravation of fibrosis after IVB [76]. Moradian et al. [77] reported exacerbation and subsequent contracture of fibrous tissue leading to tractional retinal detachment (TRD) in 2 patients who received IVB for active aggressive PDR. In a report by Arevalo et al. [78], TRD occurred in 11 eyes (5.2%) among 211 intravitreal injections in patients with severe PDR. A longer interval between IVB and vitrectomy was among the main risk factors for the development or progression of TRD.

Histological studies have shown vascular endothelial cells to still be present in the FVMs following IVB [67, 79, 80]. No apparent fenestration was observed in newly formed vessels from the FVMs. A histological study showed apoptotic vascular endothelial cells and overexpression of smooth muscle actin in the FVMs after IVB [67]. The authors of that study concluded that IVB may induce changes in immature, newly formed vessels of PDR, leading to endothelial apoptosis with vascular regression, while inducing normalization of premature vessels by increasing the pericyte coverage and reducing vessel fenestration. Another study suggested that retinal neovascularization is markedly reduced on approximately day 10 after IVB injection, whereas contractile elements are not yet abundant [80]. Therefore, IVB may temporally reduce the blood flow of the newly formed vessels, however, it did not induce the complete regression of the vascular endothelium of the FVMs. Therefore, the blood flow of the newly formed vessels may be easily reperfused when the effect of bevacizumab diminishes. 

## 7. Retinopathy of Prematurity

An overexpression of VEGF appears to be important in the pathogenesis of retinopathy of prematurity (ROP). Laser photocoagulation of the avascular retina reduces the risk of severe visual loss, presumably by decreasing VEGF production, but causes loss of peripheral retinal function. Some patients progress to retinal detachment despite laser or cryotherapy. The functional outcomes are still not satisfying in stage 4B or 5 ROP, even after vitrectomy or scleral buckling [81, 82]. There has, therefore, been considerable interest in the potential application of anti-VEGF agents, such as bevacizumab, as primary treatment or as an adjunct to photocoagulation [83–89]. Because VEGF is highly elevated in advanced ROP and has been found to play a central role as the driving force for neovascularization [90, 91], blocking VEGF with anti-VEGF agents seems to be a reasonable approach. Bevacizumab seems to work best in stage 3 ROP. The majority of the treated eyes did not progress to stage 4 ROP even without additional injection or laser therapy. However, some patients received laser treatment if positive symptoms or neovascularization persisted or worsened [85, 92–95]. The purpose of bevacizumab use in stage 4 or 5 patients is to reduce the plus sign to help reduce hemorrhage during the subsequent vitrectomy [88, 93, 96]. Some authors reported cases of resolution of stage 4A ROP after bevacizumab injection [86, 93]. However, caution is necessary when bevacizumab is used in ROP with retinal detachment. While angiogenesis is inhibited, the fibrotic components of ROP may accelerate and retinal detachment might worsen [97]. 

 Potential local and systemic complications of intravitreal injection of anti-VEGF medications are one of the major concerns especially for infants with a very small eye size and small body mass with rapidly developing tissue [89]. VEGF plays a number of critical roles in the developing retina as a survival factor for retinal neurons [98] and in maintaining the health of the retinal pigment epithelium [99]. It also influences neuronal growth and differentiation because of its neurotrophic effects [100, 101] and has a critical role as a neuroprotectant in the central nervous system in the adaptive response to ischemia [102]. The only histopathologic study evaluating neonate ocular specimens 20 weeks after IVB showed that bevacizumab was well tolerated without any signs of toxic effects including inflammation, degeneration, or necrosis [103]. The beneficial effects and advantages of bevacizumab for ROP that have been reported in the limited literature to date, indicate that further randomized control trials are warranted. 

## References

[1] Rosenfeld PJ, Brown DM, Heier JS (2006). Ranibizumab for neovascular age-related macular degeneration. *The New England Journal of Medicine*.

[2] Brown DM, Michels M, Kaiser PK, Heier JS, Sy JP, Ianchulev T (2009). Ranibizumab versus verteporfin photodynamic therapy for neovascular age-related macular degeneration: two-year results of the ANCHOR study. *Ophthalmology*.

[3] Regillo CD, Brown DM, Abraham P (2008). Randomized, double-masked, sham-controlled trial of ranibizumab for neovascular age-related macular degeneration: PIER study year 1. *American Journal of Ophthalmology*.

[4] Abraham P, Yue H, Wilson L (2010). Randomized, double-masked, sham-controlled trial of ranibizumab for neovascular age-related macular degeneration: PIER study year 2. *American Journal of Ophthalmology*.

[5] Fung AE, Lalwani GA, Rosenfeld PJ (2007). An optical coherence tomography-guided, variable dosing regimen with intravitreal ranibizumab (Lucentis) for neovascular age-related macular degeneration. *American Journal of Ophthalmology*.

[6] Lalwani GA, Rosenfeld PJ, Fung AE (2009). A variable-dosing regimen with intravitreal ranibizumab for neovascular age-related macular degeneration: year 2 of the PrONTO study. *American Journal of Ophthalmology*.

[7] Chakravarthy U, Adamis AP, Cunningham ET (2006). Year 2 efficacy results of 2 randomized controlled clinical trials of pegaptanib for neovascular age-related macular degeneration. *Ophthalmology*.

[8] Friberg TR, Tolentino M (2010). Pegaptanib sodium as maintenance therapy in neovascular age-related macular degeneration: the LEVEL study. *British Journal of Ophthalmology*.

[9] Martin DF, Maguire MG, Ying G-S, Grunwald JE, Fine SL, Jaffe GJ (2011). Ranibizumab and bevacizumab for neovascular age-related macular degeneration. *The New England Journal of Medicine*.

[10] Heier JS, Boyer D, Nguyen QD (2011). The 1-year results of CLEAR-IT 2, a phase 2 study of vascular endothelial growth factor trap-eye dosed as-needed after 12-week fixed dosing. *Ophthalmology*.

[11] Campochiaro PA, Hafiz G, Shah SM (2008). Ranibizumab for macular edema due to retinal vein occlusions: implication of VEGF as a critical stimulator. *Molecular Therapy*.

[12] Spaide RF, Chang LK, Klancnik JM (2009). Prospective study of intravitreal ranibizumab as a treatment for decreased visual acuity secondary to central retinal vein occlusion. *American Journal of Ophthalmology*.

[13] Pieramici DJ, Rabena M, Castellarin AA (2008). Ranibizumab for the treatment of macular edema associated with perfused central retinal vein occlusions. *Ophthalmology*.

[14] Brown DM, Campochiaro PA, Singh RP (2010). Ranibizumab for macular edema following central retinal vein occlusion. Six-month primary end point results of a phase III study. *Ophthalmology*.

[15] Campochiaro PA, Brown DM, Awh CC (2011). Sustained benefits from ranibizumab for macular edema following central retinal vein occlusion: twelve-month outcomes of a phase III study. *Ophthalmology*.

[16] Campochiaro PA, Heier JS, Feiner L (2010). Ranibizumab for macular edema following branch retinal vein occlusion. Six-month primary end point results of a phase III study. *Ophthalmology*.

[17] Brown DM, Campochiaro PA, Bhisitkul RB (2011). Sustained benefits from ranibizumab for macular edema following branch retinal vein occlusion: 12-month outcomes of a phase III study. *Ophthalmology*.

[18] Do DV, Schmidt-Erfurth U, Gonzalez VH (2011). The DA VINCI study: phase 2 primary results of VEGF trap-eye in patients with diabetic macular edema. *Ophthalmology*.

[19] Avery RL, Pearlman J, Pieramici DJ (2006). Intravitreal bevacizumab (Avastin) in the treatment of proliferative diabetic retinopathy. *Ophthalmology*.

[20] Bakri SJ, Donaldson MJ, Link TP (2006). Rapid regression of disc neovascularization in a patient with proliferative diabetic retinopathy following adjunctive intravitreal bevacizumab. *Eye*.

[21] Mason JO, Nixon PA, White MF (2006). Intravitreal injection of bevacizumab (Avastin) as adjunctive treatment of proliferative diabetic retinopathy. *American Journal of Ophthalmology*.

[22] Spaide RF, Fisher YL (2006). Intravitreal bevacizumab (Avastin) treatment of proliferative diabetic retinopathy complicated by vitreous hemorrhage. *Retina*.

[23] Wu L, Martínez-Castellanos MA, Quiroz-Mercado H (2008). Twelve-month safety of intravitreal injections of bevacizumab (Avastin): results of the pan-american collaborative retina study group (PACORES). *Graefe’s Archive for Clinical and Experimental Ophthalmology*.

[24] Avery RL (2006). Regression of retinal and iris neovascularization after intravitreal bevacizumab (avastin) treatment. *Retina*.

[25] Davidorf FH, Mouser JG, Derick RJ (2006). Rapid improvement of rubeosis iridis from a single bevacizumab (avastin) injection. *Retina*.

[26] Kahook MY, Schuman JS, Noecker RJ (2006). Intravitreal bevacizumab in a patient with neovascular glaucoma. *Ophthalmic Surgery Lasers and Imaging*.

[27] Grisanti S, Biester S, Peters S, Tatar O, Ziemssen F, Bartz-Schmidt KU (2006). Intracameral bevacizumab for Iris rubeosis. *American Journal of Ophthalmology*.

[28] Oshima Y, Sakaguchi H, Gomi F, Tano Y (2006). Regression of iris neovascularization after intravitreal injection of bevacizumab in patients with proliferative diabetic retinopathy. *American Journal of Ophthalmology*.

[29] Iliev ME, Domig D, Wolf-Schnurrbursch U, Wolf S, Sarra GM (2006). Intravitreal bevacizumab (Avastin) in the treatment of neovascular glaucoma. *American Journal of Ophthalmology*.

[30] Yazdani S, Hendi K, Pakravan M (2007). Intravitreal bevacizumab (Avastin) injection for neovascular glaucoma. *Journal of Glaucoma*.

[31] Mason JO, Albert MA, Mays A, Vail R (2006). Regression of neovascular iris vessels by intravitreal injection of bevacizumab. *Retina*.

[32] Yazdani S, Hendi K, Pakravan M, Mahdavi M, Yaseri M (2009). Intravitreal bevacizumab for neovascular glaucoma: a randomized controlled trial. *Journal of Glaucoma*.

[33] Rizzo S, Genovesi-Ebert F, Bartolo E, Vento A, Miniaci S, Williams G (2008). Injection of intravitreal bevacizumab (Avastin) as a preoperative adjunct before vitrectomy surgery in the treatment of severe proliferative diabetic retinopathy (PDR). *Graefe’s Archive for Clinical and Experimental Ophthalmology*.

[34] Schmidinger G, Maar N, Bolz M, Scholda C, Schmidt-Erfurth U (2011). Repeated intravitreal bevacizumab (AvastinÂ) treatment of persistent new vessels in proliferative diabetic retinopathy after complete panretinal photocoagulation. *Acta Ophthalmologica*.

[35] Wakabayashi T, Oshima Y, Sakaguchi H (2008). Intravitreal bevacizumab to treat iris neovascularization and neovascular glaucoma secondary to ischemic retinal diseases in 41 consecutive cases. *Ophthalmology*.

[36] Saito Y, Higashide T, Takeda H, Murotani E, Ohkubo S, Sugiyama K (2010). Clinical factors related to recurrence of anterior segment neovascularization after treatment including intravitreal bevacizumab. *American Journal of Ophthalmology*.

[37] Takihara Y, Inatani M, Kawaji T (2011). Combined intravitreal bevacizumab and trabeculectomy with mitomycin C versus trabeculectomy with mitomycin C alone for neovascular glaucoma. *Journal of Glaucoma*.

[38] Saito Y, Higashide T, Takeda H, Ohkubo S, Sugiyama K (2010). Beneficial effects of preoperative intravitreal bevacizumab on trabeculectomy outcomes in neovascular glaucoma. *Acta Ophthalmologica*.

[39] Chen E, Park CH (2006). Use of intravitreal bevacizumab as a preoperative adjunct for tractional retinal detachment repair in severe proliferative diabetic retinopathy. *Retina*.

[40] Ishikawa K, Honda S, Tsukahara Y, Negi A (2009). Preferable use of intravitreal bevacizumab as a pretreatment of vitrectomy for severe proliferative diabetic retinopathy. *Eye*.

[41] Da Lucena DR, Ribeiro JAS, Costa RA (2009). Intraoperative bleeding during vitrectomy for diabetic tractional retinal detachment with versus without preoperative intravitreal bevacizumab (IBeTra study). *British Journal of Ophthalmology*.

[42] Peters S, Heiduschka P, Julien S (2007). Ultrastructural findings in the primate eye after intravitreal injection of bevacizumab. *American Journal of Ophthalmology*.

[43] Marneros AG, Fan J, Yokoyama Y (2005). Vascular endothelial growth factor expression in the retinal pigment epithelium is essential for choriocapillaris development and visual function. *American Journal of Pathology*.

[44] Roberts WG, Palade GE (1995). Increased microvascular permeability and endothelial fenestration induced by vascular endothelial growth factor. *Journal of Cell Science*.

[45] Dastjerdi MH, Al-Arfaj KM, Nallasamy N (2009). Topical bevacizumab in the treatment of corneal neovascularization; results of a prospective, open-label, noncomparative study. *Archives of Ophthalmology*.

[46] Koenig Y, Bock F, Horn F, Kruse F, Straub K, Cursiefen C (2009). Short- and long-term safety profile and efficacy of topical bevacizumab (Avastin) eye drops against corneal neovascularization. *Graefe’s Archive for Clinical and Experimental Ophthalmology*.

[47] Vassileva PI, Hergeldzhieva TG (2009). Avastin use in high risk corneal transplantation. *Graefe’s Archive for Clinical and Experimental Ophthalmology*.

[48] Aiello LP, Avery RL, Arrigg PG (1994). Vascular endothelial growth factor in ocular fluid of patients with diabetic retinopathy and other retinal disorders. *The New England Journal of Medicine*.

[49] Pe’er J, Folberg R, Itin A, Gnessin H, Hemo I, Keshet E (1998). Vascular endothelial growth factor upregulation in human central retinal vein occlusion. *Ophthalmology*.

[50] Moss SE, Klein R, Klein BEK (1994). Ten-year incidence of visual loss in a diabetic population. *Ophthalmology*.

[51] Paulus YM, Gariano RF (2009). Diabetic retinopathy: a growing concern in an aging population. *Geriatrics*.

[52] Klein R, Knudtson MD, Lee KE, Gangnon R, Klein BEK (2009). The Wisconsin Epidemiologic Study of Diabetic Retinopathy XXIII: the twentyfive- year incidence of macular edema in persons with type 1 diabetes. *Ophthalmology*.

[53] Nguyen QD, Shah SM, Van Anden E, Sung JU, Vitale S, Campochiaro PA (2004). Supplemental oxygen improves diabetic macular edema: a pilot study. *Investigative Ophthalmology and Visual Science*.

[54] Vinores SA (1997). Upregulation of vascular endothelial growth factor in ischemic and non-ischemic human and experimental retinal disease. *Histology and Histopathology*.

[55] Derevjanik NL, Vinores SA, Xiao WH (2002). Quantitative assessment of the integrity of the blood-retinal barrier in mice. *Investigative Ophthalmology and Visual Science*.

[56] Ozaki H, Hayashi H, Vinores SA, Moromizato Y, Campochiaro PA, Oshima K (1997). Intravitreal sustained release of VEGF causes retinal neovascularization in rabbits and breakdown of the blood-retinal barrier in rabbits and primates. *Experimental Eye Research*.

[57] Massin P, Bandello F, Garweg JG (2010). Safety and efficacy of ranibizumab in diabetic macular edema (RESOLVE study): a 12-month, randomized, controlled, double-masked, multicenter phase II study. *Diabetes Care*.

[58] Mitchell P, Bandello F, Schmidt-Erfurth U (2011). The RESTORE study: ranibizumab monotherapy or combined with laser versus laser monotherapy for diabetic macular edema. *Ophthalmology*.

[59] Nguyen QD, Shah SM, Heier JS (2009). Primary end point (Six Months) results of the ranibizumab for edema of the mAcula in diabetes (READ-2) study. *Ophthalmology*.

[60] Nguyen QD, Shah SM, Khwaja AA (2010). Two-year outcomes of the ranibizumab for edema of the mAcula in diabetes (READ-2) study. *Ophthalmology*.

[61] Johnson M, Gong H, Freddo TF, Ritter N, Kamm R (1993). Serum proteins and aqueous outflow resistance in bovine eyes. *Investigative Ophthalmology and Visual Science*.

[62] Gartner S, Henkind P (1978). Neovascularization of the iris (Rubeosis iridis). *Survey of Ophthalmology*.

[63] Kubota T, Tawara A, Hata Y, Khalil A, Inomata H (1996). Neovascular tissue in the intertrabecular spaces in eyes with neovascular glaucoma. *British Journal of Ophthalmology*.

[64] Peters S, Heiduschka P, Julien S, Bartz-Schmidt K-U, Schraermeyer U (2008). Immunohistochemical localisation of intravitreally injected bevacizumab in the anterior chamber angle, iris and ciliary body of the primate eye. *British Journal of Ophthalmology*.

[65] Ishibashi S, Tawara A, Sohma R, Kubota T, Toh N (2010). Angiographic changes in iris and iridocorneal angle neovascularization after intravitreal bevacizumab injection. *Archives of Ophthalmology*.

[66] Kubota T, Aoki R, Harada Y (2009). Trabecular meshwork in neovascular glaucoma eyes after the intravitreal injection of bevacizumab. *British Journal of Ophthalmology*.

[67] Kohno RI, Hata Y, Mochizuki Y (2010). Histopathology of neovascular tissue from eyes with proliferative diabetic retinopathy after intravitreal bevacizumab injection. *American Journal of Ophthalmology*.

[68] Sawada O, Kawamura H, Kakinoki M, Sawada T, Ohji M (2007). Vascular endothelial growth factor in aqueous humor before and after intravitreal injection of bevacizumab in eyes with diabetic retinopathy. *Archives of Ophthalmology*.

[69] Folkman J (1971). Tumor angiogenesis: therapeutic implications. *The New England Journal of Medicine*.

[70] Ferrara N (2004). Vascular endothelial growth factor: basic science and clinical progress. *Endocrine Reviews*.

[71] Adamis AP, Shima DT (2005). The role of vascular endothelial growth factor in ocular health and disease. *Retina*.

[72] Murata T, Nakagawa K, Khalil A, Ishibashi T, Inomata H, Sueishi K (1996). The relation between expression of vascular endothelial growth factor and breakdown of the blood-retinal barrier in diabetic rat retinas. *Laboratory Investigation*.

[73] Funatsu H, Yamashita H, Noma H (2005). Aqueous humor levels of cytokines are related to vitreous levels and progression of diabetic retinopathy in diabetic patients. *Graefe’s Archive for Clinical and Experimental Ophthalmology*.

[74] Wallow IHL, Geldner PS (1980). Endothelial fenestrae in proliferative diabetic retinopathy. *Investigative Ophthalmology and Visual Science*.

[75] Ahmadieh H, Shoeibi N, Entezari M, Monshizadeh R (2009). Intravitreal bevacizumab for prevention of early postvitrectomy hemorrhage in diabetic patients: a randomized clinical trial. *Ophthalmology*.

[76] Kuiper EJ, Van Nieuwenhoven FA, de Smet MD (2008). The angio-fibrotic switch of VEGF and CTGF in proliferative diabetic retinopathy. *PLoS One*.

[77] Moradian S, Ahmadieh H, Malihi M, Sohellian M, Dehgan MH, Azarmina M (2008). Intravitreal bevacizumab in active progressive in active progressive proliferative diabetic retinopathy. *Graefe's Archive for Clinical and Experimental Ophthalmology*.

[78] Arevalo JF, Maia M, Flynn HW (2008). Tractional retinal detachment following intravitreal bevacizumab (Avastin) in patients with severe proliferative diabetic retinopathy. *British Journal of Ophthalmology*.

[79] Kubota T, Morita H, Tou N (2010). Histology of fibrovascular membranes of proliferative diabetic retinopathy after intravitreal injection of bevacizumab. *Retina*.

[80] El-Sabagh HA, Abdelghaffar W, Labib AM (2011). Preoperative intravitreal bevacizumab use as an adjuvant to diabetic vitrectomy: histopathologic findings and clinical implications. *Ophthalmology*.

[81] Repka MX, Tung B, Good WV (2006). Outcome of eyes developing retinal detachment during the Early Treatment for Retinopathy of Prematurity Study (ETROP). *Archives of Ophthalmology*.

[82] Gilbert WS, Quinn GE, Dobson V, Reynolds J, Hardy RJ, Palmer EA (1996). Partial retinal detachment at 3 months after threshold retinopathy of prematurity: long-term structural and functional outcome. *Archives of Ophthalmology*.

[83] Good WV, Flynn JT, Flach AJ, Cibis GW, Raab EL, Beauchamp GR (2004). Final results of the Early Treatment for Retinopathy of Prematurity (ETROP) randomized trial. *Transactions of the American Ophthalmological Society*.

[84] Chung EJ, Kim JH, Ahn HS, Koh HJ (2007). Combination of laser photocoagulation and intravitreal bevacizumab (Avastin®) for aggressive zone I retinopathy of prematurity. *Graefe’s Archive for Clinical and Experimental Ophthalmology*.

[85] Mintz-Hittner HA, Kuffel RR (2008). Intravitreal injection of bevacizumab (avastin) for treatment of stage 3 retinopathy of prematurity in zone i or posterior zone II. *Retina*.

[86] Quiroz-Mercado H, Martinez-Castellanos MA, Hernandez-Rojas ML, Salazar-Teran N, Chan RVP (2008). Antiangiogenic therapy with intravitreal bevacizumab for retinopathy of prematurity. *Retina*.

[87] Lalwani GA, Berrocal AM, Murray TG (2008). Off-label use of intravitreal bevacizumab (Avastin) for salvage treatment in progressive threshold retinopathy of prematurity. *Retina*.

[88] Kusaka S, Shima C, Wada K (2008). Efficacy of intravitreal injection of bevacizumab for severe retinopathy of prematurity: a pilot study. *British Journal of Ophthalmology*.

[89] Micieli JA, Surkont M, Smith AF (2009). A systemic analysis of the off-label use of bevacizumab for severe retinopathy of prematurity. *American Journal of Ophthalmology*.

[90] Sato T, Kusaka S, Shimojo H, Fujikado T (2009). Vitreous levels of erythropoietin and vascular endothelial growth factor in eyes with retinopathy of prematurity. *Ophthalmology*.

[91] Nonobe NI, Kachi S, Kondo M (2009). Concentration of vascular endothelial growth factor in aqueous humor of eyes with advanced retinopathy of prematurity before and after intravitreal injection of bevacizumab. *Retina*.

[92] Nazari H, Modarres M, Parvaresh MM, Ghasemi Falavarjani K (2010). Intravitreal bevacizumab in combination with laser therapy for the treatment of severe retinopathy of prematurity (ROP) associated with vitreous or retinal hemorrhage. *Graefe’s Archive for Clinical and Experimental Ophthalmology*.

[93] Wu WC, Yeh PT, Chen SN, Yang CM, Lai CC, Kuo HK (2011). Effects and complications of bevacizumab use in patients with retinopathy of prematurity: a multicenter study in Taiwan. *Ophthalmology*.

[94] Lee JY, Chae JB, Yang SJ, Yoon YH, Kim JG (2010). Effects of intravitreal bevacizumab and laser in retinopathy of prematurity therapy on the development of peripheral retinal vessels. *Graefe’s Archive for Clinical and Experimental Ophthalmology*.

[95] Hoang QV, Kiernan DF, Chau FY, Shapiro MJ, Blair MP (2010). Fluorescein angiography of recurrent retinopathy of prematurity after initial intravitreous bevacizumab treatment. *Archives of Ophthalmology*.

[96] Kychenthal A, Dorta P (2010). Vitrectomy after intravitreal bevacizumab (Avastin) for retinal detachment in retinopathy of prematurity. *Retina*.

[97] Honda S, Hirabayashi H, Tsukahara Y, Negi A (2008). Acute contraction of the proliferative membrane after an intravitreal injection of bevacizumab for advanced retinopathy of prematurity. *Graefe’s Archive for Clinical and Experimental Ophthalmology*.

[98] Saint-Geniez M, Maharaj ASR, Walshe TE (2008). Endogenous VEGF is required for visual function: evidence for a survival role on Müller cells and photoreceptors. *PLoS One*.

[99] Blaauwgeers HGT, Holtkamp GM, Rutten H (1999). Polarized vascular endothelial growth factor secretion by human retinal pigment epithelium and localization of vascular endothelial growth factor receptors on the inner choriocapillaris: evidence for a trophic paracrine relation. *American Journal of Pathology*.

[100] Schwarz Q, Gu C, Fujisawa H (2004). Vascular endothelial growth factor controls neuronal migration and cooperates with SemaSA to pattern distinct compartments of the facial nerve. *Genes and Development*.

[101] Sondell M, Lundborg G, Kanje M (1999). Vascular endothelial growth factor has neurotrophic activity and stimulates axonal outgrowth, enhancing cell survival and Schwann cell proliferation in the peripheral nervous system. *Journal of Neuroscience*.

[102] Nishijima K, Ng YS, Zhong L (2007). Vascular endothelial growth factor-A is a survival factor for retinal neurons and a critical neuroprotectant during the adaptive response to ischemic injury. *American Journal of Pathology*.

[103] Kong L, Mintz-Hittner HA, Penland RL, Kretzer FL, Chévez-Barrios P (2008). Intravitreous bevacizumab as anti-vascular endothelial growth factor therapy for retinopathy of prematurity: a morphologic study. *Archives of Ophthalmology*.

